# RETRACTION: Effect of Chinese Herbal Topical Medicine, Acupuncture, and Moxibustion on Pressure Ulcer Wound Healing: A Meta‐Analysis

**DOI:** 10.1111/iwj.70417

**Published:** 2025-03-26

## Abstract

**RETRACTION:** WangF. , ChenM. , and DuJ. , “Effect of Chinese Herbal Topical Medicine, Acupuncture, and Moxibustion on Pressure Ulcer Wound Healing: A Meta‐Analysis,” International Wound Journal 19, no. 8 (2022): 2031–2038, 10.1111/iwj.13803.35396823 PMC9705186

The above article, published online on 09 April 2022, in Wiley Online Library (http://onlinelibrary.wiley.com/), has been retracted by agreement between the journal Editor in Chief, Professor Keith Harding; and John Wiley & Sons Ltd. Following an investigation by the publisher, all parties have concluded that this article was accepted solely on the basis of a compromised peer review process. The editors have therefore decided to retract the article. The authors did not respond to our notice regarding the retraction.

**RETRACTION:** F. Wang , M. Chen , and J. Du , “Effect of Chinese Herbal Topical Medicine, Acupuncture, and Moxibustion on Pressure Ulcer Wound Healing: A Meta‐Analysis,” International Wound Journal 19, no. 8 (2022): 2031–2038, 10.1111/iwj.13803.35396823 PMC9705186

The above article, published online on 09 April 2022, in Wiley Online Library (http://onlinelibrary.wiley.com/), has been retracted by agreement between the journal Editor in Chief, Professor Keith Harding; and John Wiley & Sons Ltd. Following an investigation by the publisher, all parties have concluded that this article was accepted solely on the basis of a compromised peer review process. The editors have therefore decided to retract the article. The authors did not respond to our notice regarding the retraction.

